# Ultrasound localization microscopy to image and assess microvasculature in a rat kidney

**DOI:** 10.1038/s41598-017-13676-7

**Published:** 2017-10-20

**Authors:** Josquin Foiret, Hua Zhang, Tali Ilovitsh, Lisa Mahakian, Sarah Tam, Katherine W. Ferrara

**Affiliations:** Department of Biomedical Engineering, University of California, Davis, California, USA

## Abstract

The recent development of ultrasound localization microscopy, where individual microbubbles (contrast agents) are detected and tracked within the vasculature, provides new opportunities for imaging the vasculature of entire organs with a spatial resolution below the diffraction limit. In stationary tissue, recent studies have demonstrated a theoretical resolution on the order of microns. In this work, single microbubbles were localized *in vivo* in a rat kidney using a dedicated high frame rate imaging sequence. Organ motion was tracked by assuming rigid motion (translation and rotation) and appropriate correction was applied. In contrast to previous work, coherence-based non-linear phase inversion processing was used to reject tissue echoes while maintaining echoes from very slowly moving microbubbles. Blood velocity in the small vessels was estimated by tracking microbubbles, demonstrating the potential of this technique to improve vascular characterization. Previous optical studies of microbubbles in vessels of approximately 20 microns have shown that expansion is constrained, suggesting that microbubble echoes would be difficult to detect in such regions. We therefore utilized the echoes from individual MBs as microscopic sensors of slow flow associated with such vessels and demonstrate that highly correlated, wideband echoes are detected from individual microbubbles in vessels with flow rates below 2 mm/s.

## Introduction

Our overall goal is to develop a safe and non-invasive tool to characterize blood flow within capillary scale vessels. Contrast-enhanced ultrasound has now been utilized for decades to image the vascularization and perfusion of tissues through the use of ultrasound contrast agents. This technique relies on injecting microbubbles (MBs) of gas that will mix in blood and freely circulate within the vasculature. Their non-linear response to an ultrasound field provides the ability to separate their echoes from those generated by surrounding tissue by using dedicated sequences of pulse transmissions. Destruction-replenishment strategies, in which a high-pressure pulse is first used to disrupt the MBs, have been proposed to map perfusion in diseases affecting the heart^1^, kidneys^2^ and peripheral vasculature^3^ or in cancer^4,5^. Similarly, the time to replenishment of the signal intensity following the destruction of MBs was also investigated in diseased tissues, with the goal to detect impaired microvascular perfusion^6,7^. However, the elimination of high amplitude pulses, which are required to destroy MBs, is desirable as biological effects can result from such pulses under limited circumstances^8^.

The recent development of ultrafast ultrasound systems offers the opportunity to capture images at a high frame rate using planar insonation, where the time required for pulse propagation to the region of interest ultimately dictates the frame rate^9^. Multiple applications have benefited from such systems, e.g. ultrafast Doppler imaging of red blood cell motion was introduced to improve detection and sensitivity to blood flow; however, these methods cannot be used to image capillary scale vessels in moving tissues^10^. Ultrafast imaging of MBs has also emerged as a new method to image and characterize blood flow, with greater sensitivity and enhanced safety^11–14^.

Optical super localization techniques, such as photoactivated localization microscopy (PALM)^15^ or stochastic optical reconstruction microscopy (STORM)^16^, have significantly impacted fluorescence microscopy, improving spatial resolution by an order of magnitude. These methods rely on the activation and precise localization of individual photo-switchable fluorophores to provide sub-diffraction resolution. Inspired by these localization methods, applications in ultrasound imaging using single MBs as acoustic point sources have recently been proposed^17,18^. If the concentration of contrast agents in the blood stream is low enough to resolve the returning echoes, MBs appear as individual scatterers with a spatial dimension determined by the point spread function (PSF) of the imaging system. Localizing the center of each MB on a sub-wavelength grid facilitates reconstructing an image of the vasculature, by creating a density map of the MB positions after recording a large number of detections. A theoretically-achievable resolution on the order of a few microns for clinical ultrasound frequencies and imaging arrays has been demonstrated^19^, and has been hence referred to as ultrasound localization microscopy (ULM). The first studies of ULM have employed a traditional clinical imaging system with limited temporal resolution, and required a low MB concentration and a long acquisition period^18^. Recently, the application of ultrafast imaging to ULM (uULM) overcame this limitation, and successfully imaged microvasculature using high MB concentrations and low mechanical index to limit MB disruption^17,20^. MB detection is usually performed using amplitude and modulation schemes^21,22^, although a spatiotemporal clutter filter scheme^23^ has also been proposed. Using uULM, *in vivo* images of the microvasculature, with penetration depth of a few centimeters and improved acquisition time, were achieved.

However, imaging with sub-diffraction resolution presents challenges *in vivo*, especially for tissues or organs undergoing physiological motion such as respiration. Over the past years, *in vivo* ULM studies have primarily focused on static tissue such as an immobilized mouse ear^18^ or a rat head in a stereotaxic frame^17^. This immobilization was required, since for capillary scale vessels, the physiological motion can be several orders of magnitude larger than the imaging resolution. For most abdominal imaging applications, the acquisition time of a few minutes using a high-frame rate system will require correction for physiological motion present in the stack of recorded images. Although the high frame rate maximizes the correlation between successive frames, the presence of MBs brings rapid decorrelation in the sequence, especially in highly vascularized organs, which renders accurate motion compensation challenging in acquisitions requiring several minutes. Phase correlation was recently applied to uULM acquired from the rat brain to correct for motion, assuming a rigid translation but not in a constraint-free environment^24^.

In this work, we report on ULM applied in the rat kidney microvasculature *in vivo* under free-breathing to investigate the effects of physiological motion and to investigate MB echoes within small vessels. This is an ideal system to develop and validate this technique since the anatomy and physiology are well established. Historical invasive measurements of red blood cell velocity in the descending and ascending vasa recta demonstrated mean red blood cells velocities of 1.04 ± 0.10 and 0.38 ± 0.03 mm/sec, individual vessel diameters of 15.6 ± 0.5 and 20.0 ± 0.4 µm, respectively and these vessels are packaged into packed bundles that are on the order of 100 µm in the inner stripe^25,26^. These red cell velocities are similar to or smaller than the surrounding tissue motion (>1 mm/s). We therefore focus on developing the techniques required to quantify these velocities non-invasively. A motion compensation method is applied to the detected MB positions to correct for changes in kidney position during the acquisition, considering a rigid in-plane motion that includes both translation and rotation. Tracking of individual MBs within small vessels is employed, and we segment the echoes to study vessels with a velocity below 2 mm/s^27^. We have previously demonstrated by optical imaging that MB expansion is constrained in vessels of similar or smaller diameters, and therefore it is also important to understand the properties of MB echoes in this vessel size range^28^. The resulting methods provide an enhanced tool for characterizing the microvasculature and improved tools to characterize microbubble oscillation within the constraints of the microvasculature.

## Results

**Figure 1:**
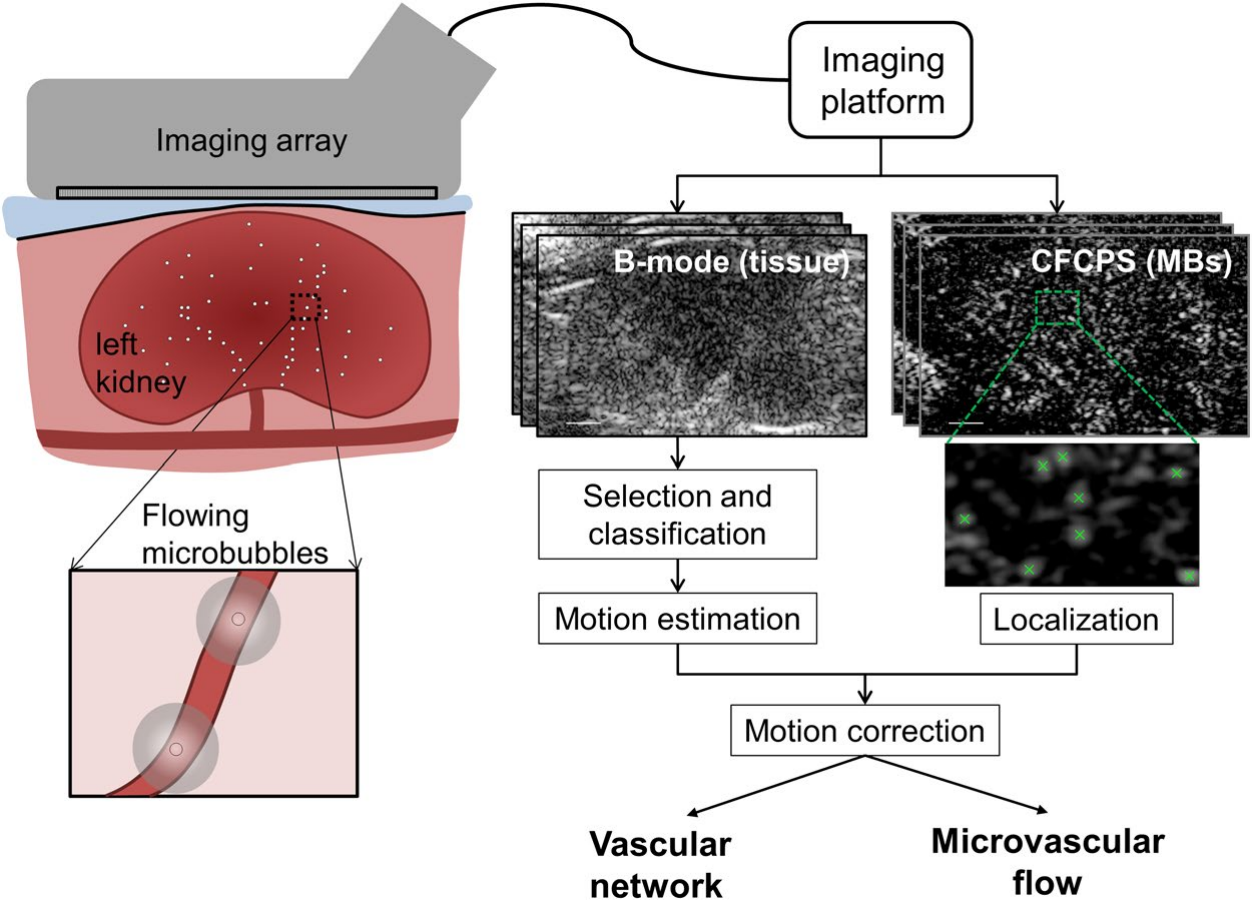
Flowchart of the acquisition and processing. Imaging of the rat kidney was performed under anesthesia (free breathing) using a compact clinical imaging array transmitting at 6.9 MHz (note that the imaging array and the kidney are not drawn to scale). An ultrasound research platform was used to acquire a stack of 40000 frames at 300 Hz, with a dedicated sequence outputting both regular B-mode images and microbubble specific Coherence Factor Contrast Pulse Sequencing (CFCPS) images. Processing of the stack allowed us to track and correct for the organ motion (B-mode) and to localize and follow the microbubble positions over time (CFCPS). After correction of the MB positions, combining all of the localizations reveals the microvascular network and associated flow.

We set out to detect capillary scale velocity (~1 mm/s) in 20 µm vessels within the vasa recta. The individual vessels within a bundle are immediately adjacent to one another^26^ and therefore cannot be resolved; individual bundles in the medulla are separated by distances on the order of 400 µm. Using our ultrasound frequency of 6.9 MHz, aperture of 22.8 mm, and imaging depth of 10 mm, the diffraction-limited resolution is approximately 110 µm axially, 330 µm laterally (in-plane), and 700 µm in elevation (out-of-plane). After a bolus injection of MBs into the tail vein, the kidney was imaged with plane waves at a high frame rate (300 Hz), and 40000 image frames were recorded. Frame acquisition used a nonlinear pulsing scheme known as contrast pulse sequencing (CPS), which is designed to differentiate MB echoes from those of the surrounding tissue^21^. Thus, three successive single cycle ultrasound pulses with varied phase and amplitude (½, −1, ½) were directed to each of 3 different angles (−5°, 0°, 5°), and one full frame was the combination of 9 transmit and receive events. The echo amplitude of the CPS images was mapped according to the Coherence Factor (CFCPS) to generate higher signal to noise ratio (SNR) images of the echoes from individual MBs. Each frame generated an image of the MB position and a separate B-Mode tissue image (Fig. 1), and MB trajectories were then visible within the resulting images (see video in Supplementary Fig. S1).

### Detecting physiological motion

**Figure 2:**
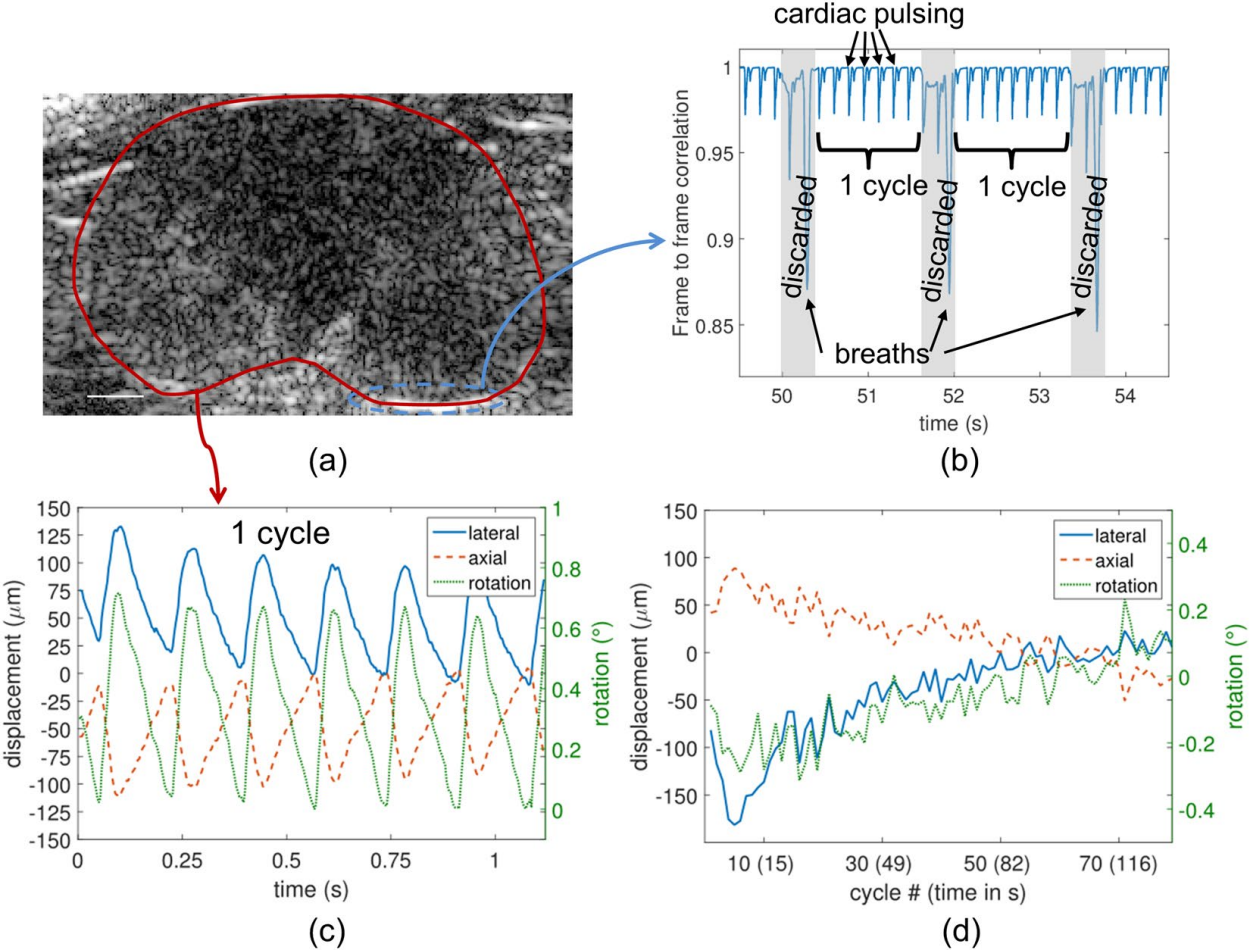
Motion detection and classification of the frames. (**a**) B-mode images were used to select frames and classify them in cycles and to detect physiological motion of the kidney. (**b**) Selection and classification of frames was performed using frame-to-frame correlation over a region-of-interest (blue dashed line) showing specular reflection and displayed here over a small time window. Respiratory motion was detected when decorrelation was prominent and associated frames were discarded from further analysis. Frames recorded between 2 successive breaths were grouped as one cycle. Small intra-cycle changes in correlation are related to cardiac pulsation from the descending aorta. (**c**) The intra-cycle motion (axial and lateral translation and rotation) over the entire kidney as a function of time shows the cyclic cardiac pulsing. (**d**) The inter-cycle motion shows the small changes of the kidney position between breaths (cycle 50 used as the reference).

Cross-correlation between successive frames was employed to track kidney motion (Fig. 2). Throughout the sequence, detection of both respiratory and cardiac-induced motion appeared as a decrease in correlation (Fig. 2(a),(b)). 79 respiratory cycles were recorded which led us to select 27833 frames out of the dataset (30% rejection). The in-plane respiratory motion reached approximately 1.5 mm and was prominently observed in the lateral direction (Supplementary Fig. S1). The average number of frames per cycle was 350, corresponding to 1.17 s of recording.

Estimation of intra-cycle motion clearly showed cyclic cardiac pulsing resulting in in-plane displacements smaller than 125 µm (Fig. 2(c)) with a range of 120 ± 8 µm and 148 ± 19 µm for axial and lateral motions, respectively. Inter-cycle motion (Fig. 2(d)) also showed small changes in kidney position between breaths. It should be noted that a small rotation was always present both intra- and inter-cycle, validating the choice of a rigid motion model for the kidney motion. The displacements were much smaller than the elevation focus of the imaging array (see Supplementary Fig. S2), and therefore small out-of-plane motions should not have significantly impacted the result.

### Image of the microvasculature

**Figure 3:**
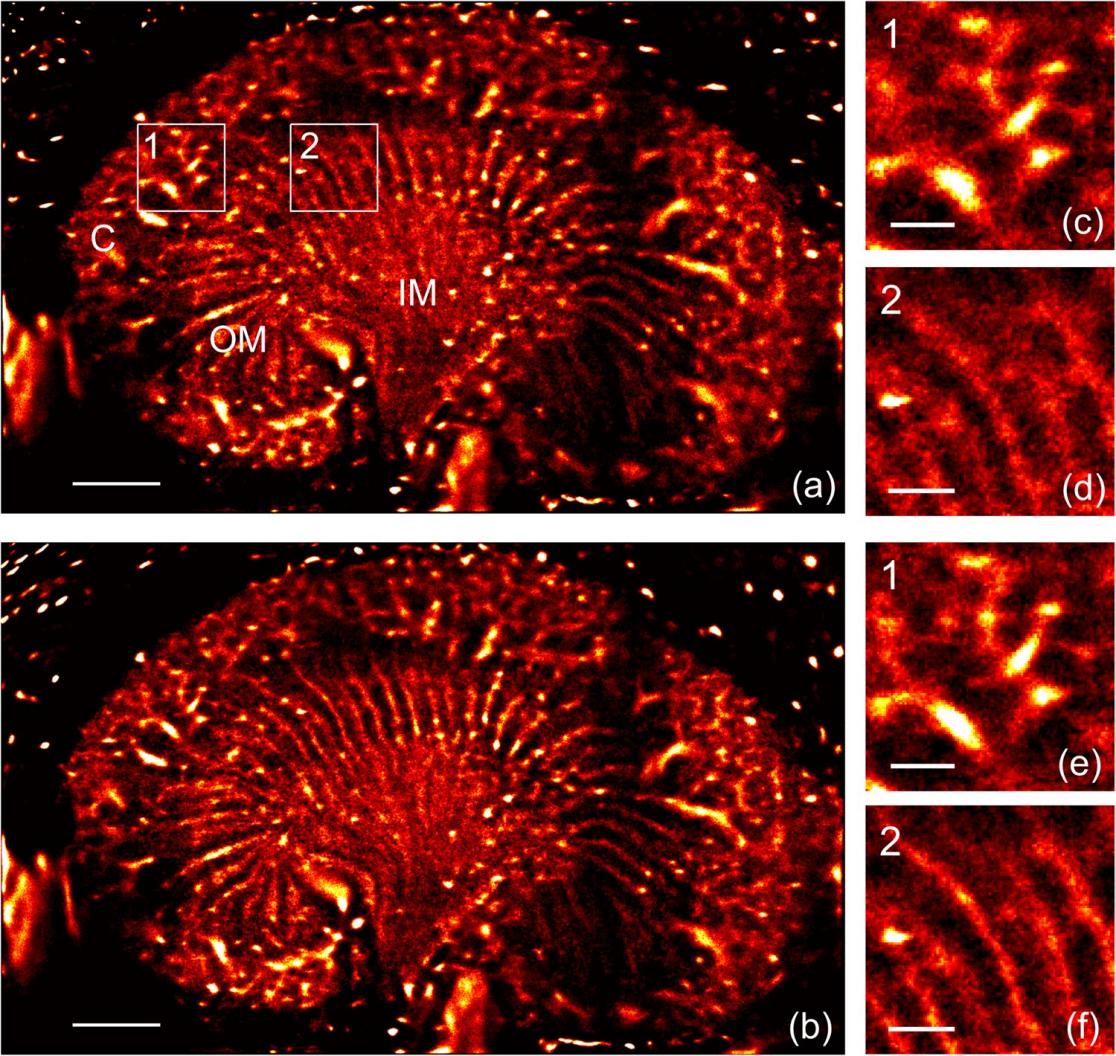
Image of the rat kidney microvasculature. (**a**), (**b**) Density map of the microbubbles positions without (**a**) and with (**b**) motion correction revealing the different vascular structures in the kidney (C: cortex, OM: outer medulla, IM: inner medulla). (**c**–**f**) Zoom over the distinctive cortex and outer medulla network without (**c**), (**d**) and with (**e**), (**f**) motion correction. Scale bar represents 2 mm in (**a**), (**b**) and 500 µm in (**c**–**f**).

In the rat kidney, approximately 3.9 million MB positions were detected over the selected stack of frames resulting in a detailed image of the in-plane kidney vasculature (Fig. 3(a),(b)). Consistent with the kidney structure, different vascular patterns were observed between the cortex and the inner and outer medulla. Long, regularly spaced vascular bundles consistent with the position of the vasa recta were observed in the outer medulla in agreement with optical observations^29^. The average size and spacing was 110 ± 33 µm (−3 dB value) and 400 ± 45 µm respectively, although the individual capillaries within the bundles could not be resolved. These bundles consist of a tightly spaced network of microvessels each with a diameter of 20 μm or less^25,29^. Bright spots can be detected on the image and were associated with vessels principally orientated in the elevation direction (out-of-plane). Compared with a maximum intensity projection image of the stack, which results in an average bundle size of 354 ± 46 µm, ULM demonstrates at least a 3-fold improvement in the accuracy of the vessel diameter measurement and facilitates resolution of vessels separated by a distance smaller than elevation beam width (Supplementary Fig. S3).

The applied motion compensation reduced the effect of changes in kidney position and improved the localization of MBs (Supplementary Fig. S4). The improvement was noticeable in the medulla (where vessels are aligned with the imaging plane) when comparing the density map of positions without (Fig. 3(a),(c) and (d)) and with (Fig. 3(b),(e) and (f)) compensation. Without compensation, the average size of the bundle increased to 144 ± 45 µm without a significant change in the bundle spacing (Supplementary Fig. S4), indicating that the motion correction improves the reconstruction accuracy by ~25%.

### Microvascular flow

**Figure 4:**
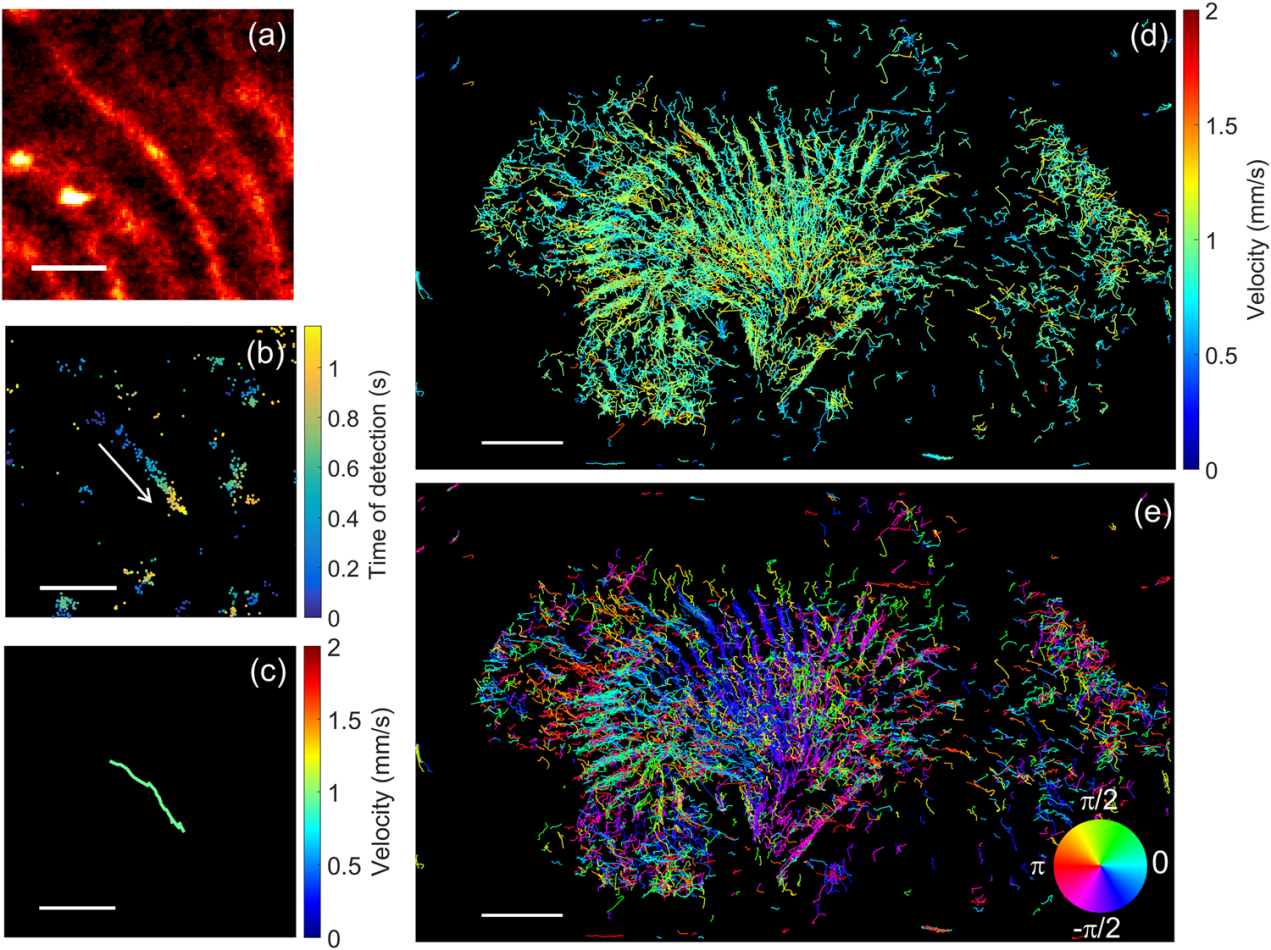
Velocity and direction of the in-plane microvascular flow represented by individual trajectories of MBs identified with a velocity smaller than 2 mm/s. (**a**) MB position density map over a region of interest (ROI). (**b**) In the same ROI and for a single cycle, the positions of the MBs are displayed as a function of time (colorbar: time of arrival in s). (**c**) Reconstructed average trajectory and velocity from the data in (**b**) (colorbar: velocity in mm/s). Trajectory was reconstructed using a nearest-neighbor method. (**d**) All of the trajectories with a velocity smaller than 2 mm/s detected for the dataset (colorbar: velocity in mm/s). The vast majority of the trajectories were found in the medulla. (**e**) Average direction of the blood flow in the microvascular network. The cyclic colorbar indicates the trajectory direction in radians. Most trajectories in the medulla are found to follow the descending vasa recta (outer to inner medulla). Scalebar represents 500 µm in (**a**), (**b**) and (**c**) and 2 mm in (**d**) and (**e**).

We find that estimation of the velocity of MB transport is feasible in small vessels such as those shown in Fig. 4(a). The arrival time of the MB in successive pixels was mapped and visualized as a pseudocolored overlay, and this arrival time was used to estimate the local velocity of the MB (Fig. 4(b)). Application of these methods in *in vitro* experiments in small fibers with controlled MB flow (Supplementary Fig. S5) produced accurate estimates of mm/s velocities and served as validation for the flow estimation. Furthermore, the estimated diameter for the fiber of 150 µm (−10 dB of the normalized cross-section) is consistent with the established laminar flow (yielding to more detection in the center of the fiber).

Here, for flow velocities smaller than 2 mm/s in vessels on the order of 20 µm, we observed MBs that could be tracked over 1 s (Fig. 4(b)). Tracking over greater time intervals is limited by motion induced by the respiratory cycle. The distinct isolated traces of these slowly flowing MBs facilitated the recovery of their trajectory and velocity within the vasculature, with an example of a MB moving within a small vessel shown in (Fig. 4(c)). A total of 4748 trajectories were recovered, principally localized in the medulla (Fig. 4(d)). The mean trajectory length detected for MBs with this range of flow rates was 500 ± 220 µm with a mean duration of 0.55 ± 0.25 s. By tracking the arrival time, as in Fig. 4(b), the direction of the MB motion was also determined. In the medulla, the majority of the MBs flowing at this low velocity were detected moving from the outer to the inner region likely as part of the descending vasa recta (Fig. 4(e)). The mean velocity in the vascular bundles of the medulla was 0.94 ± 0.24 mm/s, which is consistent with the reported values from previous invasive studies^25^. Fewer trajectories were detected in the cortex and in large vessels as a result of the maximum velocity threshold of 2 mm/s applied here. The difference arises from the higher blood velocities and resulting decrease in detection due to the 40 successive time point requirement.

### Microbubble signature in small vessels

**Figure 5:**
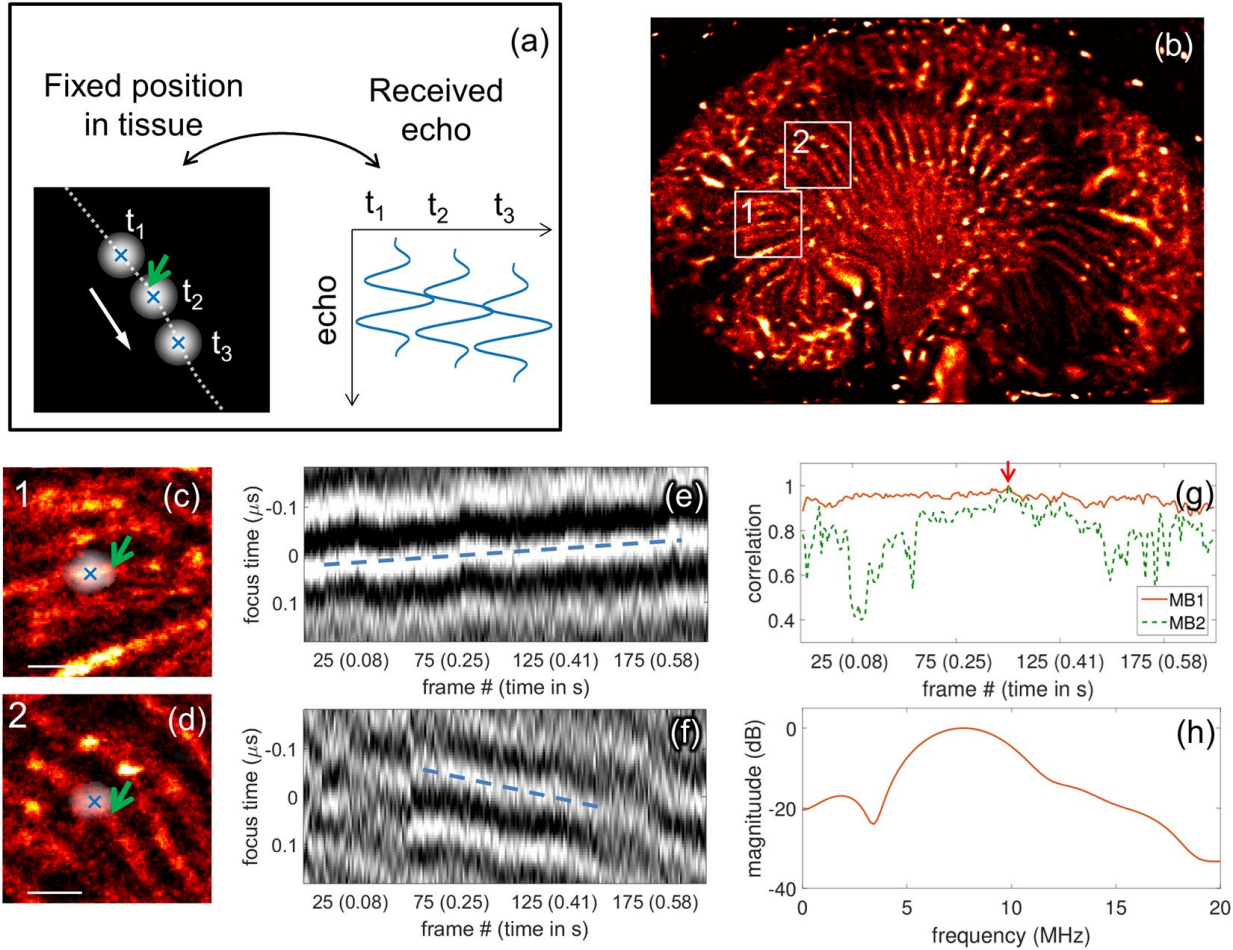
Echoes from individual MBs can be tracked over hundreds of frames in small vessels. (**a**) Diagram of the microbubble motion and the echoes after alignment to remove the trend in position. (**b**) Density map showing the location of the regions of interest (ROI) displaying vessels with slow blood flow. (**c**), (**d**), shows a combined view of the ROI with a CFCPS frame superimposed showing an isolated MB (appearing as the PSF of the imaging system) that was tracked over numerous frames, where the scale bar represents 500 µm. (**e**), (**f**), Time traces of the tracked MB as a function of the frame number. The focus time indicates the fixed position in tissue along the MB path after correcting for tissue motion. For reference, at 6.9 MHz (transmit frequency) one period equals 0.14 µs. (**g**) Correlation of the echoes over a large number of frames is evidenced if a single echo is used as a reference (indicated by the red arrow). (**h**) Average spectrum of the traces displayed in (**e**) with a peak at the fundamental transmission frequency.

Tracking individual MBs also provided the opportunity to quantify the spectrum and correlation of the echoes over time (Fig. 5). As shown in Fig. 5(a), for MBs with a velocity below 2 mm/s, the composite set of echoes from an individual MB was detected and aligned to correct for tissue motion. As described above, the image of the moving MB is blurred by the finite transducer bandwidth and diffraction, therefore, the position of the MB was estimated (cross overlay) based on the processed and aligned echoes, providing the super-resolved estimate of MB position. By following the MBs in small vessels on the CFCPS frames and using the corresponding aligned received time signals, the MB positions (white overlap corresponds to blurred MB image and cross overlay corresponds to detected MB center location) shown in Fig. 5(c) and (d) were visualized. The successive echoes from an individual MB were then displayed as a radiofrequency image (similar in format to an M-mode ultrasound image), with an overlay in blue for the center of the MB, such that the correlation between successive echoes can be directly visualized (Fig. 5(e),(f)). We found that echoes from slowly flowing MBs remained correlated over hundreds of frames, where this time period (blue line overlay) was likely limited by their passage through the elevational beam width or their disruption. The highly-correlated transit time interval was approximately 200 frames (1800 pulses acquired over 0.7 second) in Fig. 5(e), and 100 frames (900 pulses acquired over 0.4 second) in Fig. 5(f). Further, the high correlation for the MB echoes in Fig. 5(e),(f) is quantified in Fig. 5(g). This observation suggests two things: first that MBs in small vessels can survive thousands of ultrasound pulses, and second, that the MB echo frequency and decorrelation rate could be used to further characterize MB oscillation within the vasculature. Here, the spectrum of the received echoes was centered on the transmission frequency (Fig. 5(h)), but contains additional high frequency spectral components in agreement with other findings^30^. Thus, here, by segmenting MBs with a very slow flow rate, we are able to demonstrate that MBs within small vessels continue to create effective wideband echoes that can be detected from a commercial transducer.

**Figure 6:**
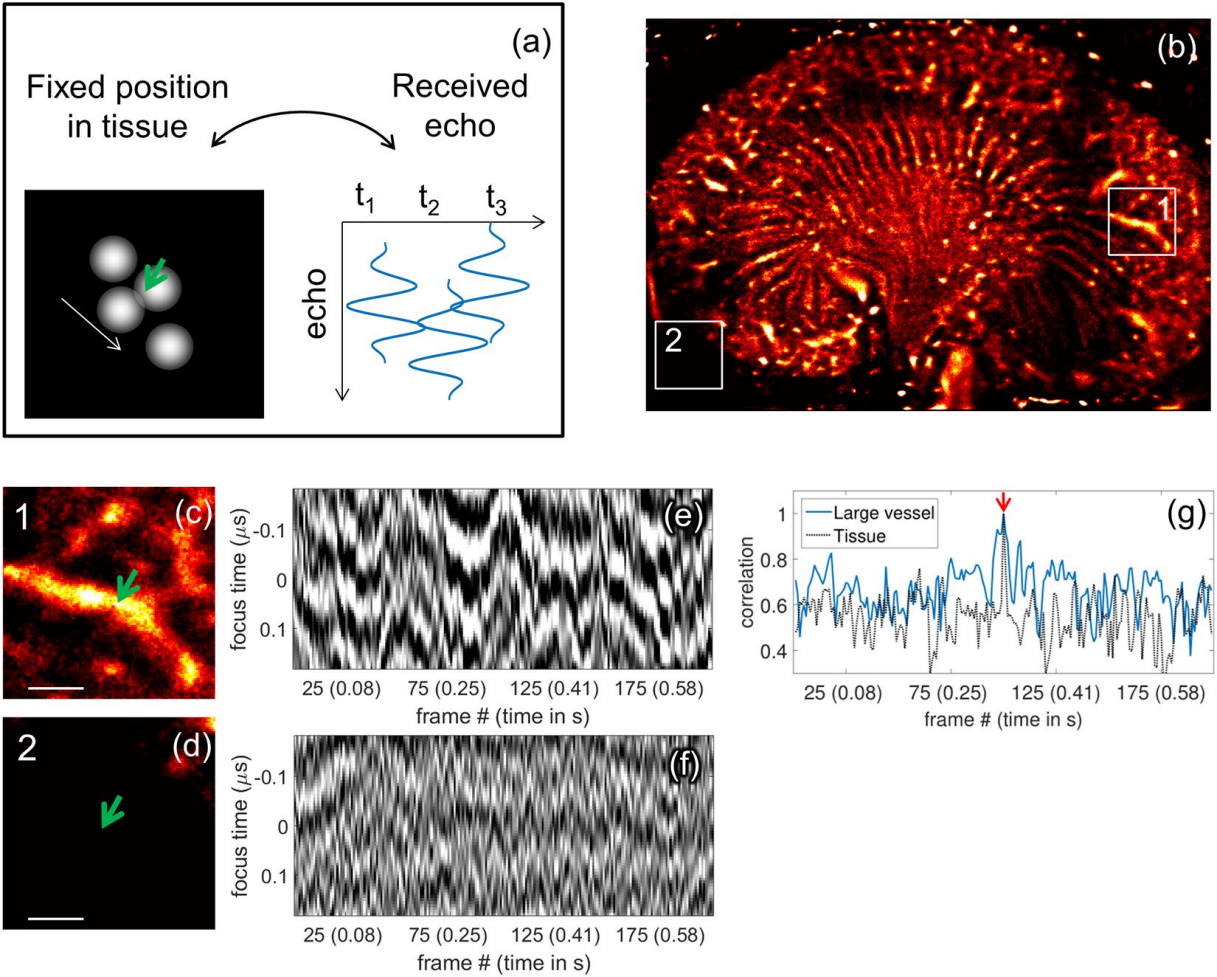
By comparison with isolated MBs, tissue background or larger vessels do not remain correlated over a large number of frames. (**a**) Diagram of the microbubble motion and the echoes that are shown in this figure in grey scale after alignment. (**b**) Density map showing the location of the regions of interest over a larger vessel (**c**) and outside of the kidney (**d**). In (**c**) and (**d**), the arrow indicates the position where time traces are visualized and the scale bar represents 500 µm. (**e**) Short-time correlation (over a few frames) is found in the vessels characterizing faster MB velocities and (**f**) noise is observed over time in the tissue. In (**g**), fast decorrelation of the echoes is evidenced if a single echo is used as a reference (indicated by the red arrow).

For comparison, we also show echoes from a large vessel and from a region without apparent MBs outside the kidney (Fig. 6). The resulting echoes from a large vessel (Fig. 6(b),(c),(e)) indicate a rapid change over successive frames, resulting from fast flowing MBs and/or unresolved echoes from multiple microbubbles within a vessel, which contrasts with the small vessel observations. As a control, background signals coming from non-perfused regions (Fig. 6(d),(f)) displayed only residual noise without coherence (Fig. 6(f)). This is expected as the CPS processing has cancelled echoes from the surrounding tissue. The correlation of the echoes in Fig. 6(e) and (f) is quantified in Fig. 6(g), showing a decorrelation much faster than that of MBs in small vessels.

## Discussion

Ultrasound contrast agents provide a unique means to map microvascular flow rate, an important physiological parameter. Ultrafast imaging and tracking of individual MBs offers new ways to image the vasculature with micron resolution and using clinical ultrasound frequencies. This is a major step toward *in vivo* tissue characterization as characterization of microvasculature in deep tissue is possible. In comparison to previous work that filtered MB echoes to eliminate those that remain stationary over an extended period of time, here, we segment the MB echoes based on the nonlinear components within their echoes (using CPS) and therefore retain the very slowly moving MBs. Thus, we are studying MBs with a diameter on the order of 1 µm that are sequestered within very small blood vessels. For MBs with an axial velocity below 2 mm/s and producing a series of hundreds of correlated echoes, the MB echoes can be used to interrogate the local microvascular environment. In the absence of noise, individual MB positions and shifts in position can be accurately mapped on a spatial scale of microns. MB position was integrated over time as they moved in and out of the image plane due to their translation or tissue motion. The elevational beam width of the transducer, electronic and acoustical noise and the accuracy of tissue motion correction ultimately limited the spatial resolution of the technique.

By using optical techniques to visualize MBs in capillary tubing and exposed tissues, we have previously shown that MBs can greatly expand in a large tube. From these optical studies, we found that expansion is constrained and MB lifetime extended in vessels smaller than 20 µm^28^. In the previous experiments, we could not interrogate the echoes from MBs while watching their oscillation in small vessels. Here, this limitation was overcome and echoes were recorded from MB oscillation in small vessels. For the sequence and parameters implemented in this work, 2700 pulses/s were directed to the MBs (300 Hz pulsing for each of 3 angles and 3 phases of CPS)^30,31^ and the MBs were tracked over 1 s. The results suggest that MBs can receive thousands of pulses *in vivo* at the current ultrasound parameters and in fact, these long persisting MBs (which likely are constrained in their expansion) do provide strong and wideband echoes even in small vessels. These observations confirm our optical studies that demonstrated enhanced MB stability and lifetime in small vessels. The ability to segment MB echoes from very small vessels offers an opportunity to enhance the control and safety of ultrasound therapeutic and delivery protocols. This is of significant interest in that MB-based strategies to open the blood brain barrier (BBB) are proceeding to human translation. In those studies, MB oscillation is used to alter vascular permeability in the insonified region through mechanisms that are not yet fully clear; it also remains uncertain as to whether the BBB changes and enhanced transport resulting from MB oscillation are concentrated in large or small vessels or are associated with a wide range of vessel diameters. ULM could segment echoes from deep in the brain and provide new insights regarding the vessel diameters associated with BBB disruption. By creating maps of echoes associated with large or small vessels (using the velocity threshold to segment these vessels), MB oscillation can be interrogated within subsets of the vasculature. Validating such oscillation within the brain will be a future goal of this work.

In this work, we have applied ULM to image the rat kidney. Unlike contrast agents available in positron emission tomography or magnetic resonance imaging, MBs are an intravascular tracer and the interpretation of their echoes is therefore simplified. The images of the microvascular network and structure were consistent with the literature^29^. The medulla region, with tightly packed vessels aligned with the coronal view of the kidney, allowed us to quantify the diameter of vascular structures with a 3-fold improvement compared to the MIP image. The blood flow measured in this region was similar to reported values from historical and invasive studies of rat kidney physiology^25^. In the medulla, we detected a higher number of MBs in the descending vasa recta (DVR, from outer to inner medulla) with respect to the ascending vasa recta (AVR, from inner to outer medulla) suggesting that MB are more likely to be disrupted before reaching the AVR. We hypothesize that the disruption arises from lower blood flow in the AVR leading to longer exposure to ultrasound pulses.

In much of the MB literature, local estimation of microvascular flow rate is based on destruction-replenishment pulse sequences in which a high-pressure pulse is applied to locally fragment the MBs. The time required for their replenishment within each voxel is then estimated without segmenting individual vessels^6^. However, safety standards recommend that the mechanical index (MI), defined as the ratio of the acoustic pressure in MPa normalized by the square root of the center frequency, remain below 0.4 for MB imaging^32^ to minimize biological effects^8^. The estimation of microvessel flow rates using high frame rate imaging protocols, such as the methods described here, alleviates the requirement for a high amplitude destructive pulse. The previous destruction-replenishment method also lacks some of the unique opportunities presented by this new method, in which the directionality of flow is preserved and therefore arterial and venous flow can be differentiated. Thus, this new method extends the quantification of micro vascularization compared to time-of-arrival methods^7^, providing improved imaging of the vascular network along with direct estimates of velocity and flow direction.

### Unique features of the signal processing methods

We have previously reported on a method to create sub-resolution images using the broadband signal generated by MBs: transmission at a low frequency and reception at a higher frequency^33^. In this scenario, spatial resolution was independent of the wavelength of transmission. However, the bandwidth available with current transducer technology limits its application to imaging of deep abdominal organs. Plus, broadband echoes require rapid collapse which shortens MBs lifetime and precludes investigation of slow blood flow. The particularly small velocity (<2 mm/s) associated with flow in these micro vessels also suggest that dedicated amplitude-modulation schemes such as the CFCPS sequence proposed here may perform better than spatio-temporal filtering in these tissue regions. CPS, and other pulse inversion sequences, facilitate the detection of nonlinear features within the frequency range of the transmitted pulse. Although phase modulation techniques such as CPS cancel components of the return echoes and therefore reduce the signal, the SNR achieved here was adequate to visualize the individual MB echoes. Also, SNR improvements were obtained using coherence-based reconstruction which is effectively applied for the sparse distribution of MBs within the tissue.

### Future technology development

Here, we use MBs as microscopic sensors of individual positions rather than to resolve very closely spaced vessels. MBs, as point targets, can be applied to resolve targets separated by a distance that is limited by the experimental system, and the Cramer Rao lower bound can provide an approximate value for the theoretical resolution limit for an experimental system^19,34^. In stabilized tissue and with the setup utilized here, at a depth of 10 mm, a maximum resolution of 2.1 µm and 6.4 µm could be achieved axially and laterally, respectively. The imaging array employed in this work has a compact footprint and is intended for superficial tissue imaging in humans with an elevation focus of 0.7 mm at a depth of 10 mm (see Supplementary Fig. S2). By comparison, this elevation focus is more than an order of magnitude larger than the resolution that could be achieved with this localization technique, when applied to a single microbubble. Hence, imaging the dense three-dimensional vascular structure of the rat kidney shows the intrinsic limits of uULM with a one-dimensional array. The elevational resolution limits the resolution of individual vessels as elevation tracking information cannot be obtained. In the cortex of the rat kidney, the density and proximity of micro vessels render tracking challenging. For the same reason, it is also difficult to correct for small out-of-plane motion between successive breaths or over the duration of the acquisition. Full 2D arrays are thus appealing as they would provide direct 3D localization of the MBs.

The assumption in correcting for physiological motion was that the echo information contained in the selected frames was coming from the same imaging plane. We chose to reject frames recorded during respiration as potential out-of-plane motion could affect the observation. This is also justified by the fact that the observed in-plane kidney displacement during respiration was larger than the elevation focus. Image registration methods, which are more precise than the least-square method used in this work, including non-rigid motion compensation, will be implemented as 2D arrays become available for this application. Algorithms for handling the large 3D data sets resulting from the volumetric beam formation must then be created. Non-rigid motion compensation can then enhance the accuracy of motion correction with an ultimate benefit of improved localization and velocity estimation.

It should be noted that due to the presence of the flowing MBs, signal correlation is reduced by the MB themselves. Thus, restricting the model for motion to rigid motion (with only 3 variables) limits the number of variables and constrains the estimation and enhances localization. Potential improvements in estimation could also be achieved by isolating the tissue signal from the MB signal^35^ which could facilitate implementation of motion compensation.

Due to the difficulty of reproducing the same imaging plane between animals, the absolute microvascular and tissue velocities differ between animals. Our goal here was to demonstrate our ability to detect very slow flow in the small vessels, therefore, we summarized results from a single animal as representative of typical velocities within the rat kidney that have been previously established by invasive techniques. A future goal is to compare microvascular velocity before and after an intervention or in normal and diseased tissue within a single organ and a larger study is planned to detect these differences.

To summarize, uULM has the ability to provide unprecedented characterization of the microvasculature network and flow over entire organs while eliminating the use of high MI disruptive pulses. The sub-diffraction imaging capability offered by this method is a promising tool for ultrasound imaging but it also brings new challenges as *in vivo* applications require a precise motion compensation scheme. Our results suggest that a single microbubble within a region of very slow flow and very small vessels can be detected and used as a microscopic sensor of physiological conditions.

## Methods

### Description of the experiment and processing

Six female Sprague Dawley rats (200 g) (Charles River, Wilmington, MA) were imaged for this study. All experimental procedures were in accordance with the Guide for the Care and Use of Laboratory Animals of the National Institutes of Health (NIH), and all animal experiments were performed under a protocol approved by the Institutional Animal Care and Use Committee (IACUC) of the University of California, Davis. MBs were first prepared and injected into the tail vein through a catheter. An imaging array was positioned over the kidney to track MBs flowing in the vasculature. A high frame rate imaging sequence (300 Hz) allowing to separate MB signals from tissue signals was implemented using a research imaging platform and thousands of frames were recorded. Images of the microvascular network and flow were obtained through processing of the stack of frames.

### Microbubble preparation

MBs were made with disteroylphosphatidylcholine (DSPC), and 1,2-distearoyl-sn-glycero-3-phosphoethanolamine-N-[methoxy(polyethylene glycol)−2000] (ammonium salt) (DSPE-PEG2K) (Avanti Polar Lipids, Alabaster, AL), with a DSPC:DSPE-PEG2K of 90:10 mol/mol. MBs were prepared as reported in^36^ and briefly summarized here. First, MB precursors, which are liposome solutions, were made with a thin-film hydration method, and then kept at 4 °C until use. Before use, the liposome solutions were shaken to generate a MB suspension, which was further purified via centrifugation to remove larger (7 RCF for 1 min, followed by 16 RCF for 1 min) and smaller (300 RCF for 3 min, 3 times) MBs. The size and concentration of the purified MBs were measured with an Accusizer 770 A (Particle Sizing Systems, Port Richey, FL). Typically, MBs have a uni-modal number-weighted size distribution (1.4 ± 0.6 µm, Supplementary Fig. S6(a)), and a two-modal volume-weighted size distribution (3.2 ± 2 0.7 µm, Supplementary Fig. S6(b)). The larger MBs, with a diameter between 2 and 6 µm, although only 10% of the total MBs in number, contribute ~50% of the total volume of MBs. MBs were used for imaging within 2 hrs after final purification. For each imaging study, 7.5 × 10^6^ MBs in 150 µL of saline were injected intravenously.

### Animal preparation

Imaging was performed on the left kidney of the animal. Prior to the experiment, fur around the imaging area was shaved and then further removed using depilatory cream. During positioning and imaging, the animal was maintained under anesthesia using 1–2% isoflurane in oxygen (2 L/min) and body temperature was maintained at 37 °C. A catheter was inserted in the tail vein using a 27 G needle. The animal was placed in the supine position and the array was positioned using a 3D linear stage. Ultrasound gel was used as coupling agent. The imaging plane was optimized using B-mode and Doppler imaging as references.

### Data acquisition

All data were acquired with a programmable ultrasound system (Vantage 256, Verasonics, WA, USA) and imaging was performed with a 128-element compact linear array (CL15-7, Phillips ATL, USA; pitch: 0.18 mm, aperture: 22.8 mm, elevation focus at 10 mm depth: 0.7 mm). The center frequency for transmission was 6.9 MHz with a peak negative pressure of 250 kPa for a full amplitude pulse (corresponding to an MI of ~0.1). A dedicated Contrast Pulse Sequencing (CPS) mode^21^ with coherent compounding was implemented by sending 3 successive single cycle pulses (½ of the full amplitude was obtained by transmitting with the odd numbered elements, then a full amplitude inverted pulse was obtained by transmitting with all elements using a phase shifted excitation, and finally ½ amplitude was obtained by transmitting with the even numbered elements). In addition, coherent compounding was achieved by transmitting plane waves at 3 different angles (−5°, 0°, 5°) and one full frame was thus the combination of 9 transmit/receive events (Supplementary Fig. S7). These parameters set a diffraction-limited in-plane resolution of approximately 110 µm axially and 330 µm laterally, and an out-of-plane resolution (elevation) of 700 µm. The delay between successive transmissions was minimized with a pulse repetition frequency of 28.5 kHz and frame rate of 300 Hz. The acoustic pressure was measured in deionized degassed water using a calibrated 0.4-mm needle hydrophone (HNP-0400, Onda, Sunnyvale, CA, USA). These parameters provided an acceptable contrast-to-tissue ratio (CTR) while limiting MB destruction^11^.

A stack of 40000 frames was recorded in 133 s after the bolus injection of MBs into the tail vein of the animal. Recording started 10 s after the injection to allow the MBs to reach the microvascular network. The data processing included the following steps which are described in the following:

1)  Implementation of CPS and coherent compounding along each line of sight to reject (or maintain) tissue echoes,

2)  Image reconstruction: beam-formation to create both B-mode and CFCPS images,

3)  Motion estimation and frame rejection: calculation of motion correction requirements for intra- and inter-cycles to eliminate physiological motion (using B-mode frames),

4)  Localization of individual MBs (using CFCPS frames),

5)  Reconstruction of vascular network image: creation of a density map of MB positions after correcting for motion to reveal the vascular network,

6)  Calculation of microvascular velocity: for each cycle, track MB positions over time and create trajectories based on a nearest neighbor method and assuming a velocity ≤2 mm/s.

### CPS and B-mode image reconstruction

Due to the high frame rate, the raw radio-frequency (RF) signals were recorded during the experiment and beamforming was performed offline. All data were reconstructed and processed using Matlab (Mathworks, Natick, VA, USA). The availability of the raw RF signals provided the ability to reconstruct at the same time points both CPS images (MB) and B-mode images (tissue + MB). The summation of the RF signals (i.e. the summation of the receive matrices for each ½, −1, ½ amplitude pulses) was accomplished prior to beamforming with (½) + (−1) + (½) for CPS and (½) − (−1) + (½) for regular B-mode (Supplementary Fig. S7).

The Coherence Factor (CF), defined as the ratio of coherent intensity over incoherent intensity^37^, was here utilized on CPS data to improve the SNR of the MB echoes and is referred to as CFCPS in the following. The CF is calculated as:

1 $$CF=\frac{{\sum }_{n={n}_{1}}^{{n}_{2}}{|{\sum }_{i={M}_{1}}^{{M}_{2}}{s}_{i}(n)|}^{2}}{M{\sum }_{i={M}_{1}}^{{M}_{2}}{\sum }_{n={n}_{1}}^{{n}_{2}}|{s}_{i}{(n)}^{2}|}$$

with s_i_(n) being the time-delayed signal received at the i^th^ element at the time sample n, M = (M_2_ − M_1_) the number of elements taken into account into the summation, and T = (n_2_ − n_1_) the temporal kernel centered on the focal time. The number M was determined by the time sample (or depth) n using a constant f-number (defined as the ratio between depth and aperture) of 1.5. The temporal kernel T was chosen as 1 period (or 1 wavelength). Here, the use of plane waves allows the evaluation of the spatial coherence of the backscattered echoes over the entire imaging plane^38^. Comparison with regular delay-and-sum beamforming is given in Supplementary Fig. S8.

### Frame selection and motion estimation

The imaging array was anchored on a stage, but the duration of the acquisition meant that physiological motion was present in the recorded frames and required rejecting unusable frames and estimating in-plane motion to correct MB positions. To this end, the B-mode stack was analyzed to classify frames and estimate motion. Two sources of motion principally affected the kidney position: respiration and cardiac pulsation generated by the descending aorta. Respiration was more prominent and was detected by calculating the frame-to-frame normalized cross-correlation (CC) in a selected region of interest (ROI) over the entire stack:

2 $$CC(m)=\frac{{\sum }_{u}{\sum }_{v}IQ(u,v,m)\,.\,I{Q}^{\ast }(u,v,m+1)}{\sqrt{{\sum }_{u}{\sum }_{v}IQ(u,v,m)\,.\,I{Q}^{\ast }(u,v,m)\,.\,{\sum }_{u}{\sum }_{v}IQ(u,v,m+1)\,.\,I{Q}^{\ast }(u,v,m+1)}}$$

with IQ representing the In-phase/Quadrature data, m the time index, u and v the lateral and axial indexes within the ROI, and * denoting the complex conjugate. The ROI was defined around a specular reflection at the kidney boundary (ROI with dashed line in Fig. 2(a)) to limit decorrelation related to flowing MBs. Frames acquired during respiratory motion were detected by a drop in frame-to-frame correlation, and discarded under the assumption that the motion was large enough to induce out-of-plane motion. The remaining frames were then grouped based on their position in a respiratory cycle.

Following this selection, inter- and intra-cycle motion was estimated assuming an in-plane rigid motion (translation + rotation) of the kidney. It was assumed that for all the selected frames, the out-of-plane motion was small compared to the elevation focus of the array. Considering that a frame F_m_(u,v) is a rotated and translated replica of the reference frame F_ref_(u,v) with rotation θ_0_ and translation (u_0_, v_0_) such that:

3 $${F}_{m}(u,v)={F}_{ref}(u\,\cos \,{\theta }_{0}+v\,\sin \,{\theta }_{0}-{u}_{0},-u\,\sin \,{\theta }_{0}+v\,\cos \,{\theta }_{0}-{v}_{0})$$

the set of parameters γ = (u_0_, v_0_, θ) has to be determined. To this end, a least-square method was implemented to find the set γ_sol_ such that:

4 $${\gamma }_{sol}=\text{arg}\,\mathop{\min }\limits_{\gamma }\{{{\sum }_{u}{\sum }_{v}({F}_{m}-{F}_{ref})}^{2}\}$$

Before optimization, an ROI was defined around the kidney (ROI with solid line in Fig. 2(a)) to limit the effect of surrounding tissues and frames were interpolated to the final localization grid. The optimization was performed in Matlab using the *lsqnonlin* function of the Optimization Toolbox. Intra-cycle motion was estimated using the central frame in the cycle as the reference; inter-cycle motion was estimated using the central frame in a chosen cycle as the reference.

### Microbubble localization and velocity estimation

Detection of individual MBs was performed on selected CFCPS frames following a procedure similar to^17^. CFCPS data were first reconstructed on a 74-μm isotropic grid. After applying a Gaussian low-pass filter and interpolating to an isotropic 18.5-μm grid (4 × interpolation), MB echoes were detected using the *regionprops* function of the Image Processing Toolbox. Centroid positions were recorded based on the profile of the Gaussian-shaped point spread function and on the maximum intensity (Supplementary Fig. S9). All positions were corrected for motion using the method described in the previous section. The final density map of the MBs was constructed using weighting to compensate for the non-integer values of the pixel positions after motion correction.

In-plane blood velocity within the microvascular network was estimated by tracking MB positions over time (Supplementary Figs S10–S11). We focused on observing flow velocities smaller than 2 mm/s (corresponding to the smallest capillaries). This small velocity value facilitated tracking and a Nearest-Neighbor Method was employed. MBs that were tracked over at least 40 successive frames were analyzed. The velocity was calculated as the average distance divided by the time interval.

### Data availability

The datasets generated during and/or analysed during the current study are available from the corresponding author on reasonable request.

## Electronic supplementary material

Supplementary Information
