# Supplementary material for: Ultrasound localization microscopy to image and assess microvasculature in a rat kidney
Source: Sci Rep. 2017 Oct 20;7:13662. doi: 10.1038/s41598-017-13676-7 (PMC5651923; doi:10.1038/s41598-017-13676-7)
Supplement: Supplementary file 1 — Supplementary Information [file 41598_2017_13676_MOESM1_ESM.pdf]

Ultrasound localization microscopy to image and assess microvasculature in a rat kidney

Josquin Foiret[1], Hua Zhang[1], Tali Ilovitsh[1], Lisa Mahakian[1], Sarah Tam[1], Katherine W. Ferrara[1]

[1]Department of Biomedical Engineering, University of California, Davis, California, USA

Supplementary Information

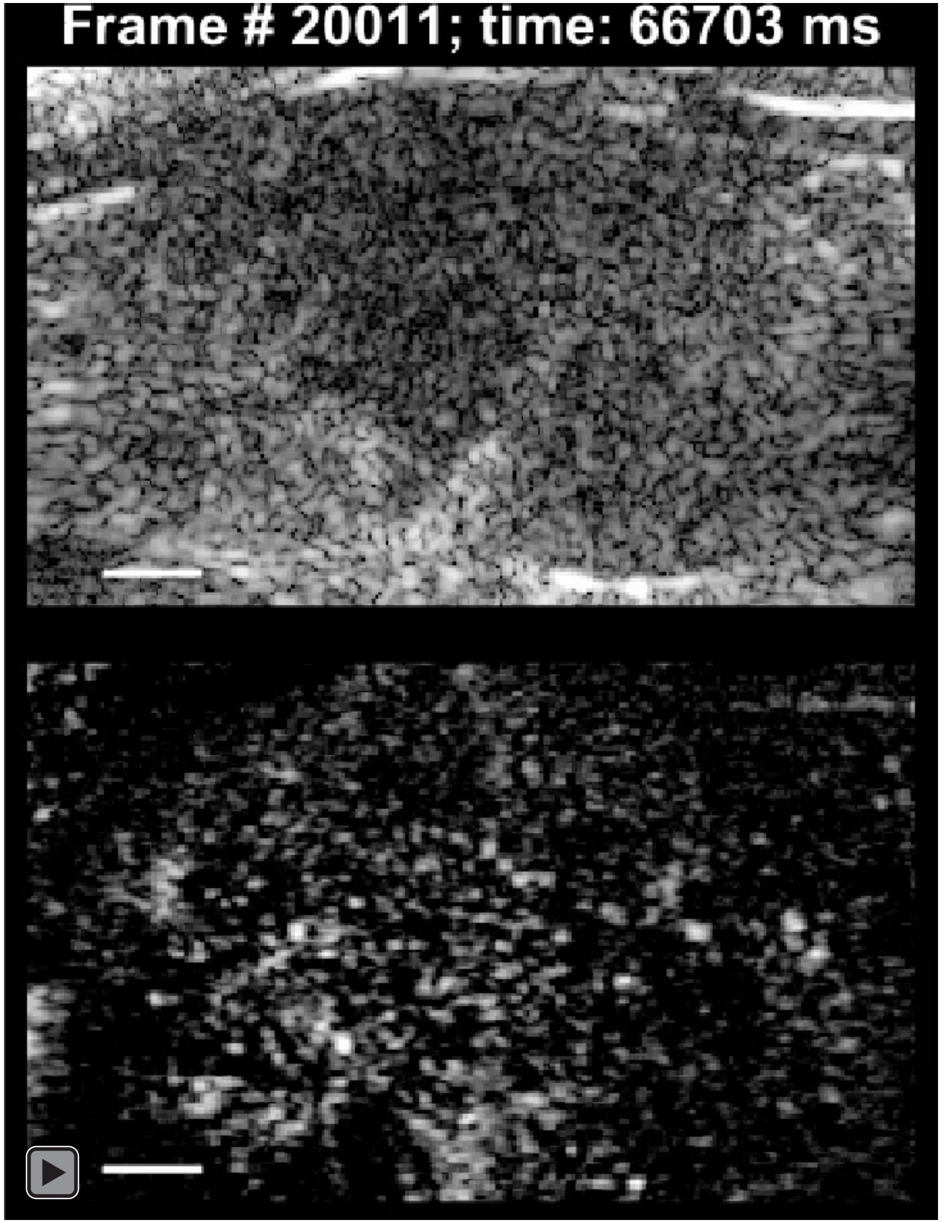

Supplementary Figure S1. Video demonstrating that physiological motion is present during the acquisition. B-mode stack (top) and CFCPS stack played at real-time framerate (dynamic range: B-mode 40 dB, CFCPS 25 dB; scale bar: 2 mm).

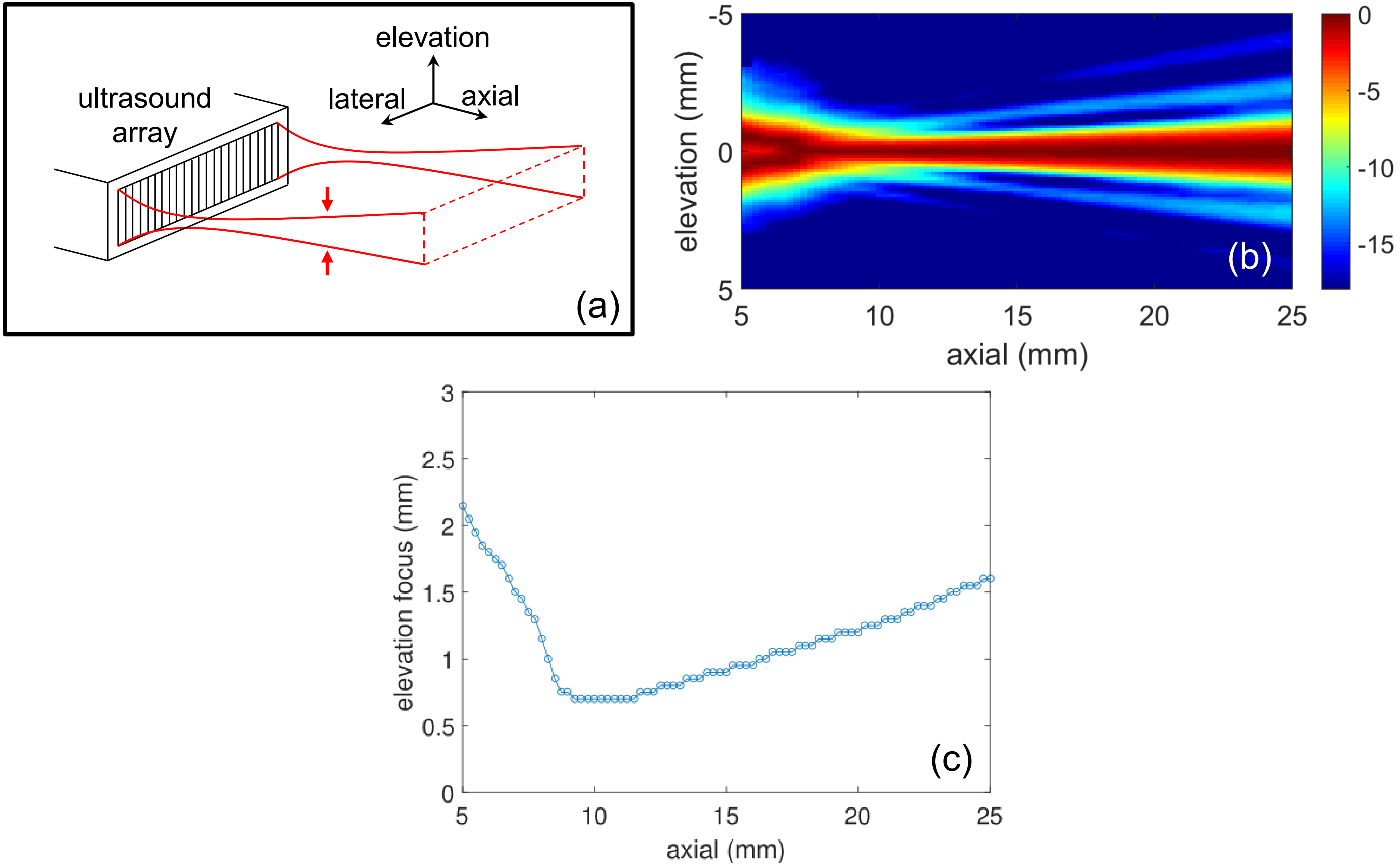

Supplementary Figure S2. Elevation resolution of the imaging array. (a) The fixed elevation focus of the imaging array sets limits for the out-of-plane resolution. (b) Intensity in the axial-elevational plane measured in water with a needle hydrophone. The intensity is displayed in dB and is normalized at each depth. (c) Full width at half maximum intensity as a function of depth. The elevation focus is minimized at ~10 mm away from the array with a full width half maximum of 0.7 mm.

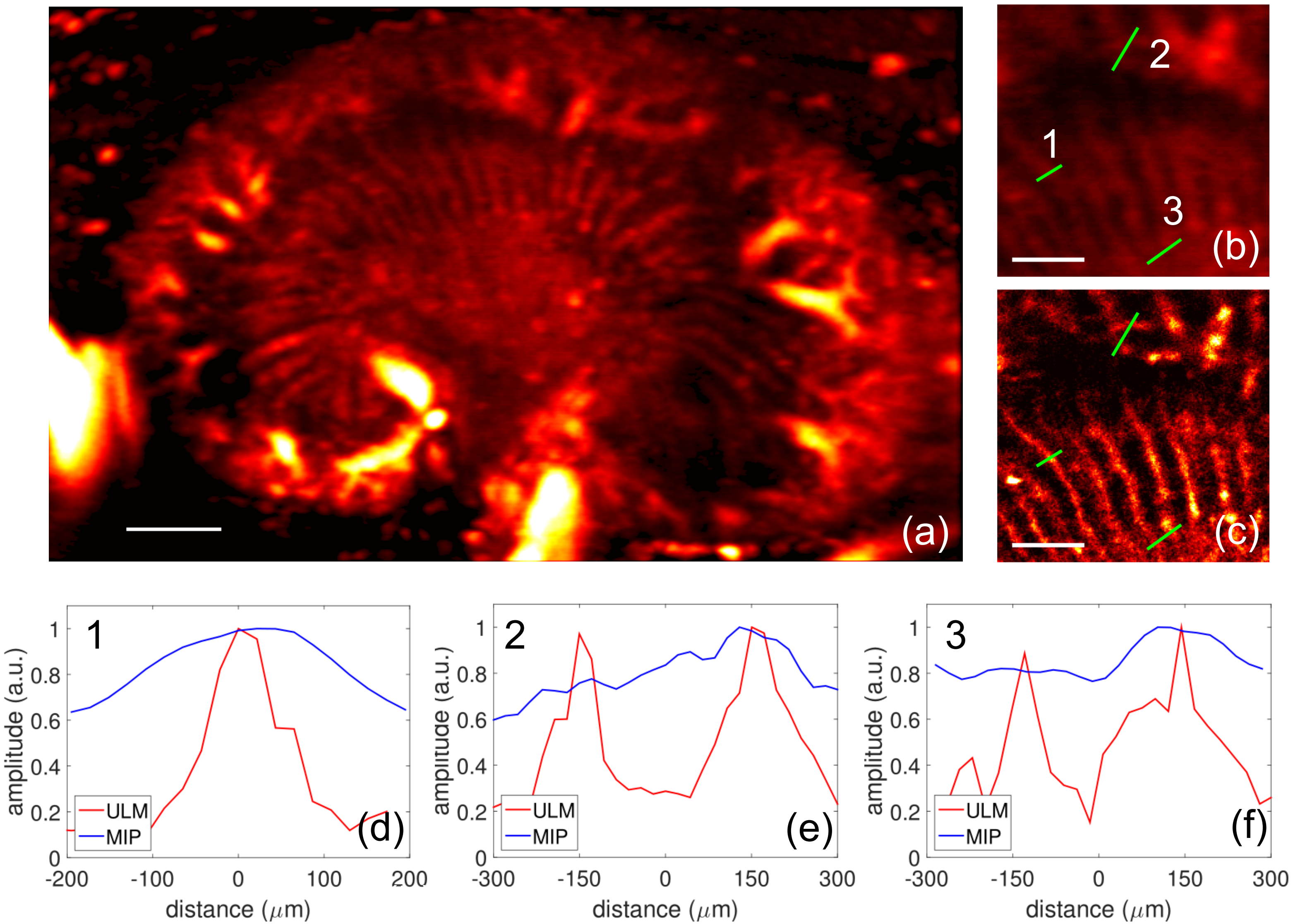

Supplementary Figure S3. Ultrasound localization microscopy improves contrast and resolution of the vasculature. (a) Maximum intensity projection (MIP) image obtained using all the frames utilized for ultrasound localization microscopy (ULM). All frames were corrected for motion. Resolution and contrast are degraded compared to ULM. (b)-(c) Zoom over the MIP image (b) and ULM image (c) indicating locations of vessels of interest (the entire ULM image is given in Figure 3). (d)-(f) Comparative cross-sections of selected vessel showing the enhanced resolution of ULM. The scale bar represents 2 mm in (a) and 1 mm in (b) and (c).

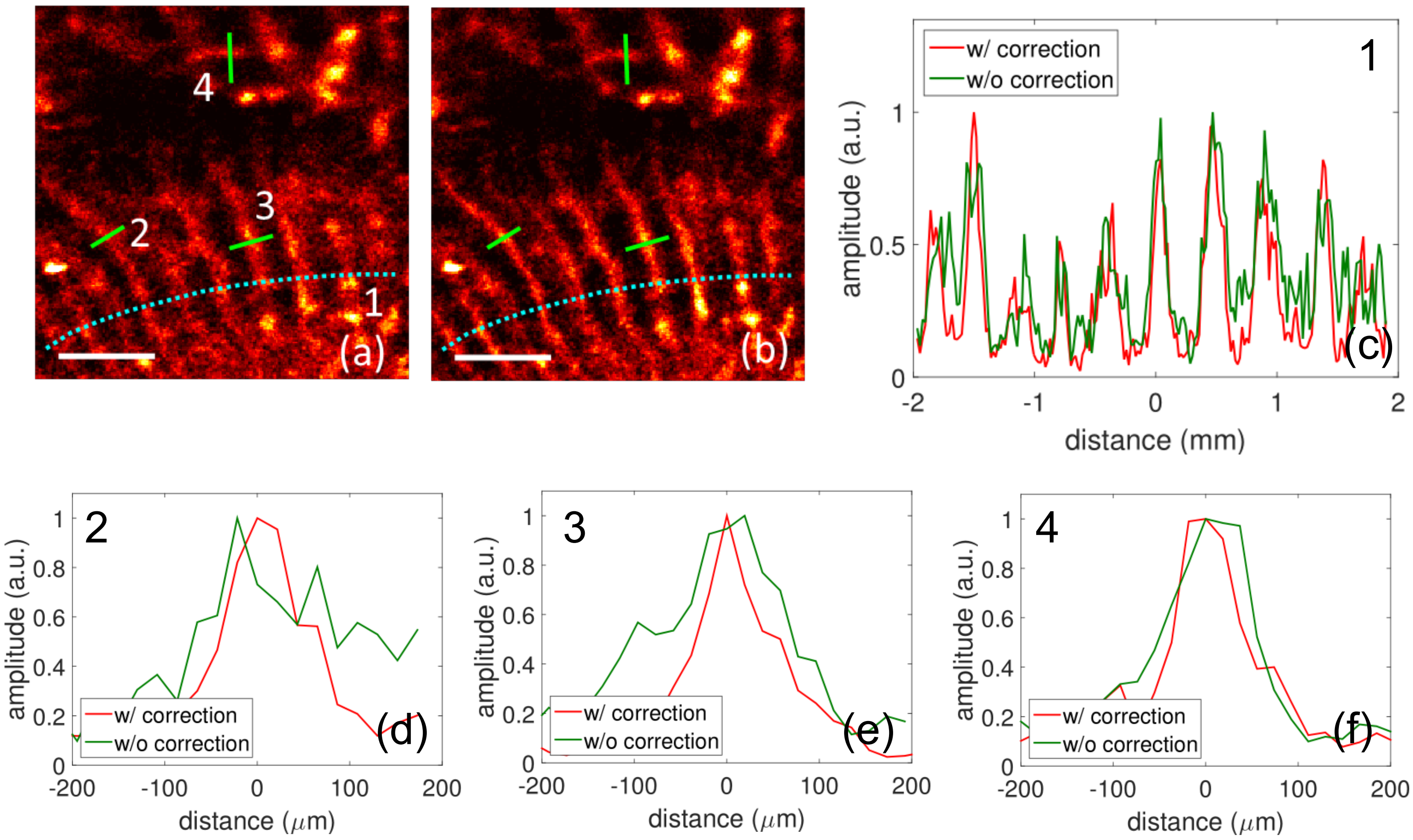

Supplementary Figure S4. Compensation for physiological motion improves imaging of the microvasculature. (a) Zoomed area over the ULM image without (a) and with (b) compensation for motion indicating locations of vessels of interest (the entire ULM image is given in Figure 3). The scale bar represents 1 mm. (c) Extended cross-section in the medulla indicating the regular spacing between vascular bundles. (d)-(f) Comparative cross-sections of selected vessels showing the enhanced resolution after motion compensation.

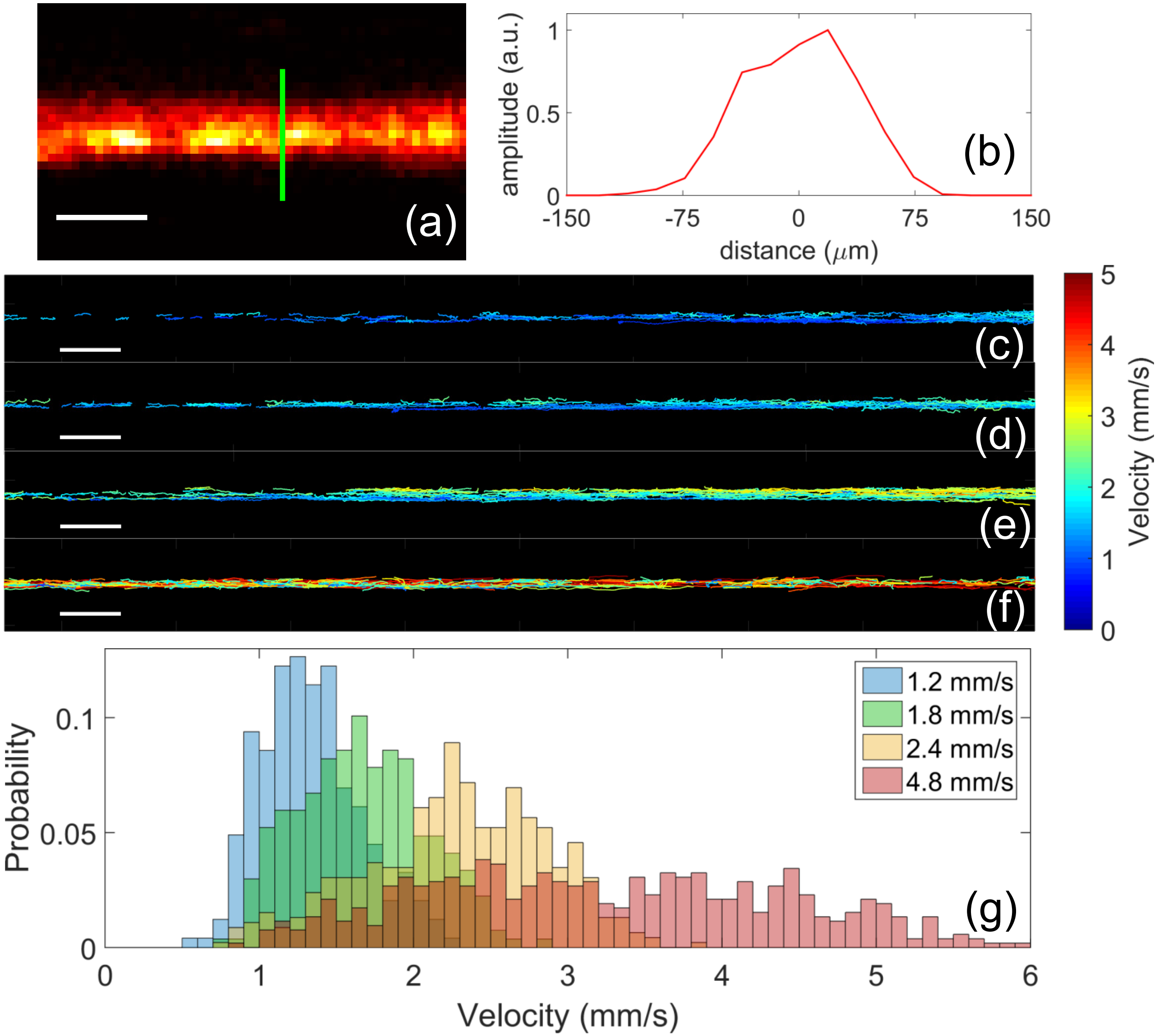

Supplementary Figure S5. *In vitro* validation of individual MB tracking to estimate the microvascular flow. Imaging was performed with MBs flowing in a 180-µm (inside diameter) cellulose fiber with a controlled and constant flow using a syringe pump. (a) Zoom over the ULM image of the fiber with a cross-section displayed in (b). The scale bar represents 200 µm (1 pixel equals 18.5 µm). (b) Cross-section shows a FWHM (-3 dB) of 100 µm and a FWTM (-10 dB) of 150 µm consistent with the laminar flow inducing more detections in the center of the fiber. (c)-(f) Trajectories recovered after tracking MBs flowing at set effective velocities of 1.2 (c), 1.8 (d), 2.4 (e) and 4.8 mm/s (f). MBs were flowing from right to left in the image. The scale bar represents 500 µm. The trajectory of each MB is color coded to its respective velocity estimate. (g) Histogram of the estimated velocities (the probability represents the count in each bin divided by the total number of counts) shows discrimination between the experiments.

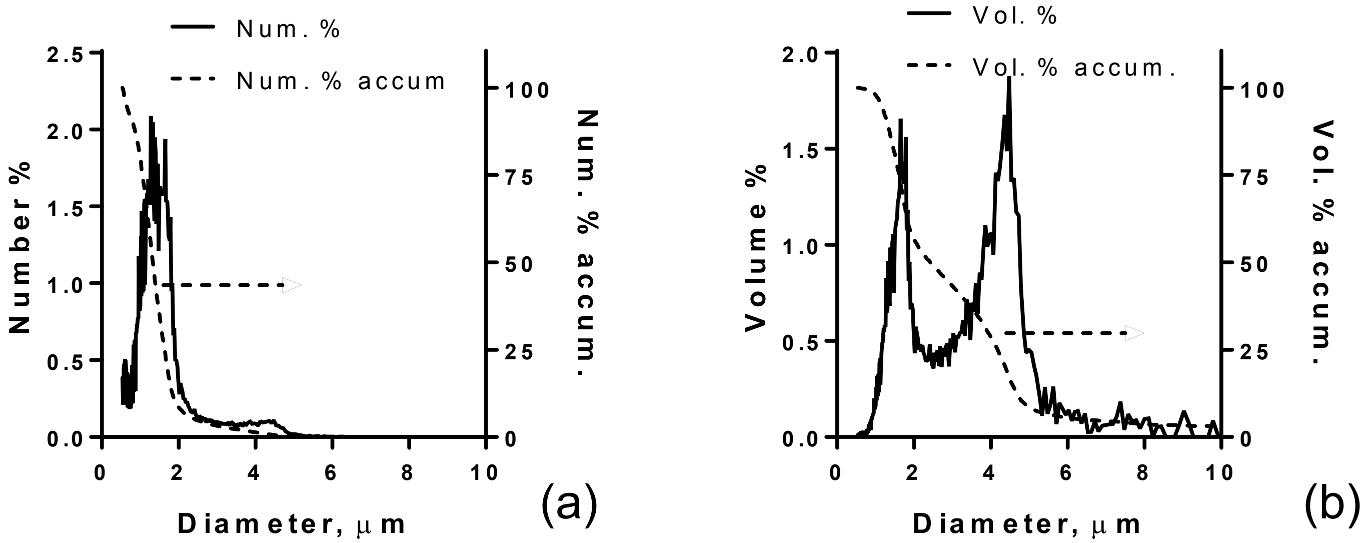

Supplementary Figure S6. Microbubble size distribution. Typical number (a), and volume (b) weighted size distributions of MBs used in this paper.

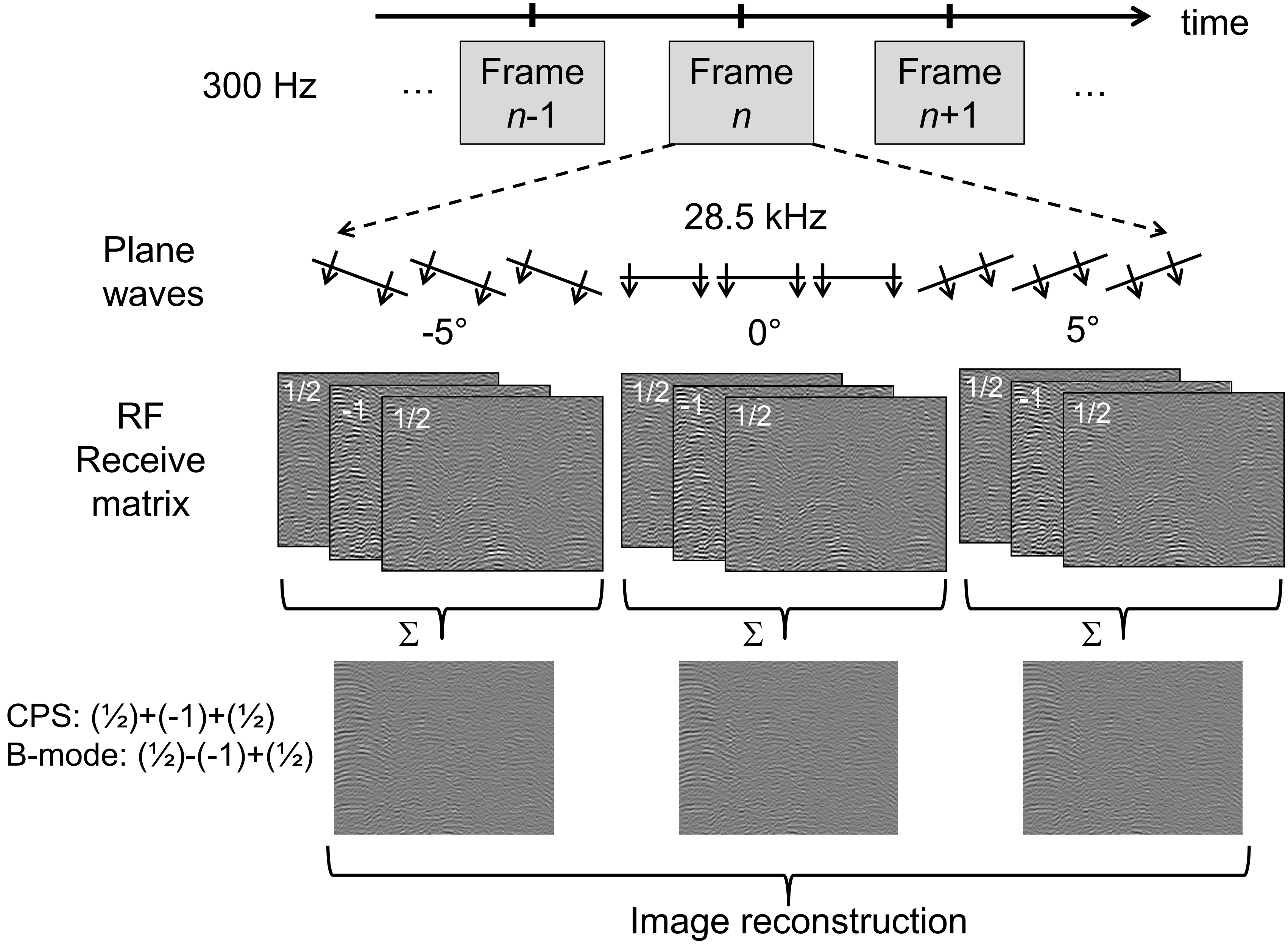

Supplementary Figure S7. Data acquisition and processing flow chart for CPS and B-mode images. Plane waves were directed to 3 angles and amplitude and phase modulated signals were combined to form the CPS matrix. The summation (i.e. the summation of the receive matrices for each ½, -1, ½ amplitude pulses) was accomplished prior to beamforming with (½)+(-1)+(½) for CPS and (½)-(-1)+(½) for regular B-mode.

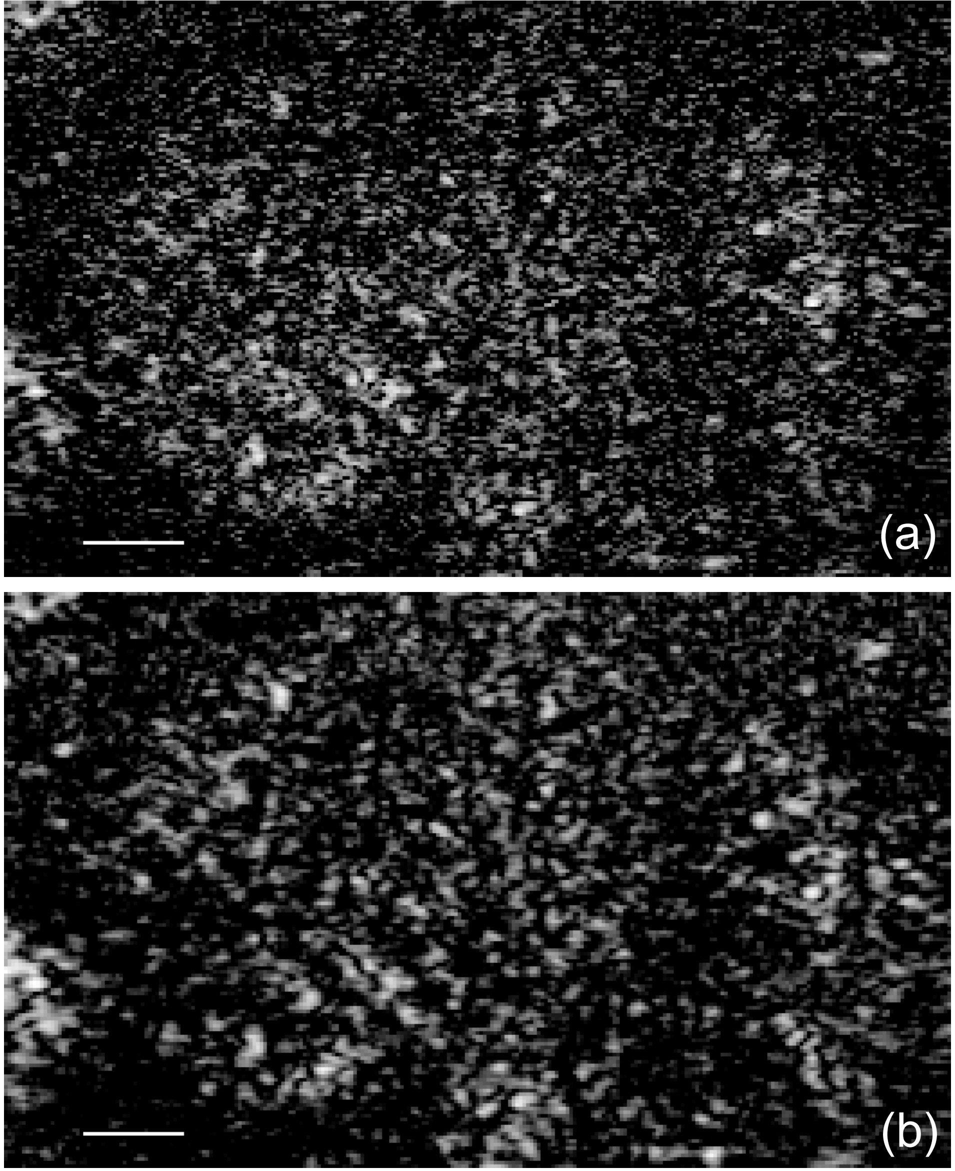

Supplementary Figure S8. Coherence factor improves detection of the MBs compared to conventional Contrast Pulse Sequencing (CPS). (a) CPS image obtained after conventional delay-and-sum beamforming and (b) Coherence Factor CPS (CFCPS) image. An f-number of 1.5 was used to reconstruct both images and the axial kernel in the coherence image was 1 wavelength. Images are displayed with a dynamic range of 25 dB. The scale bar represents 2 mm.

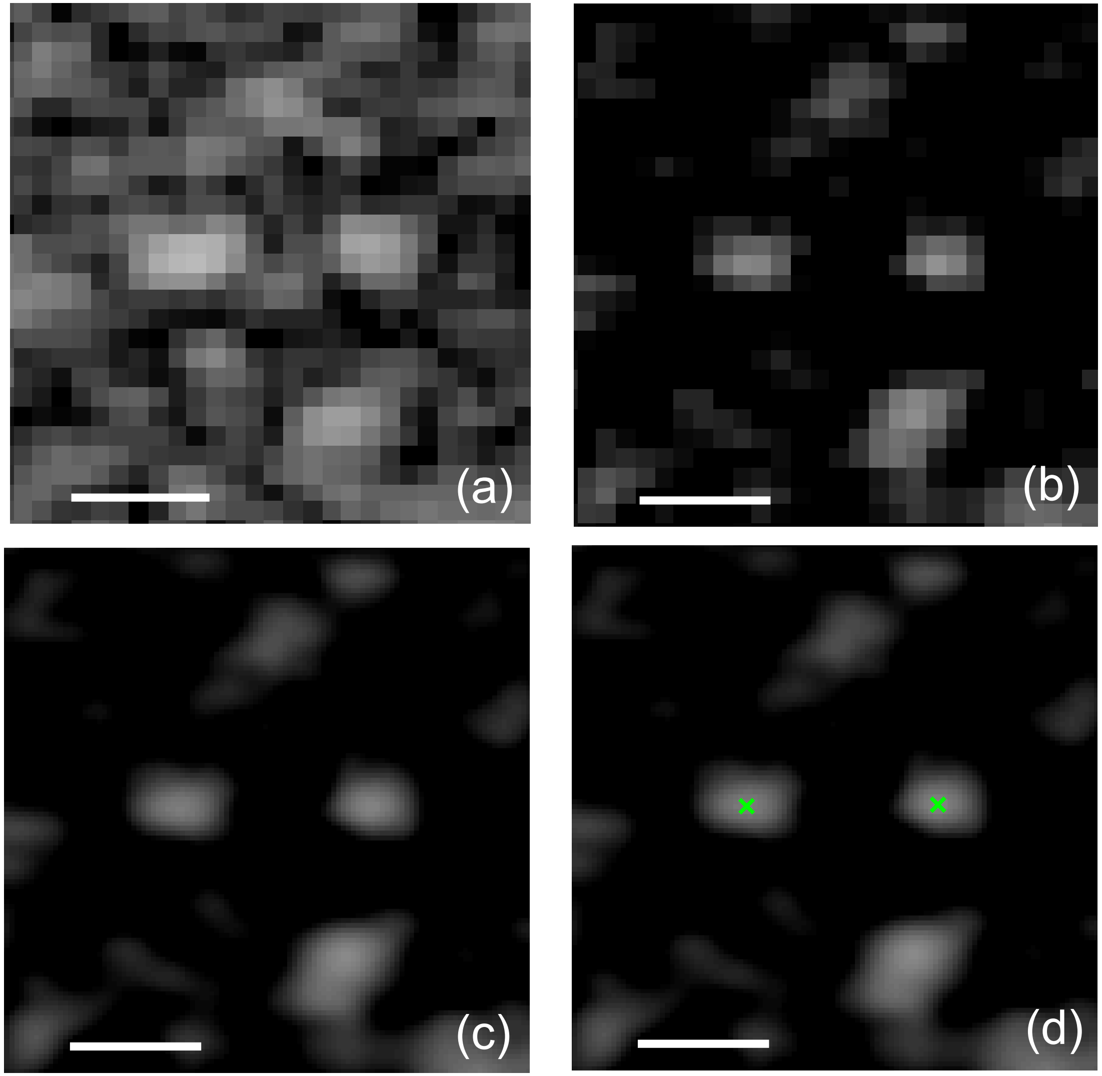

Supplementary Figure S9. Localization of individual MBs. Zoomed area from a CPS (a) and the same CFCPS (b) image showing individual MBs with pixel size of 74 µm. (c) CFCPS image after lowpass gaussian filtering and interpolation to a 18.5-µm pixel grid. (d) After application of binary masks to reject noise and/or non-separated sources, the center of the MB position is localized. The scale bar is 500 µm for all images and the dynamic range is 25 dB.

Goal: estimate the **microvascular blood flow**, *i.e.* blood velocity **<2 mm/s**

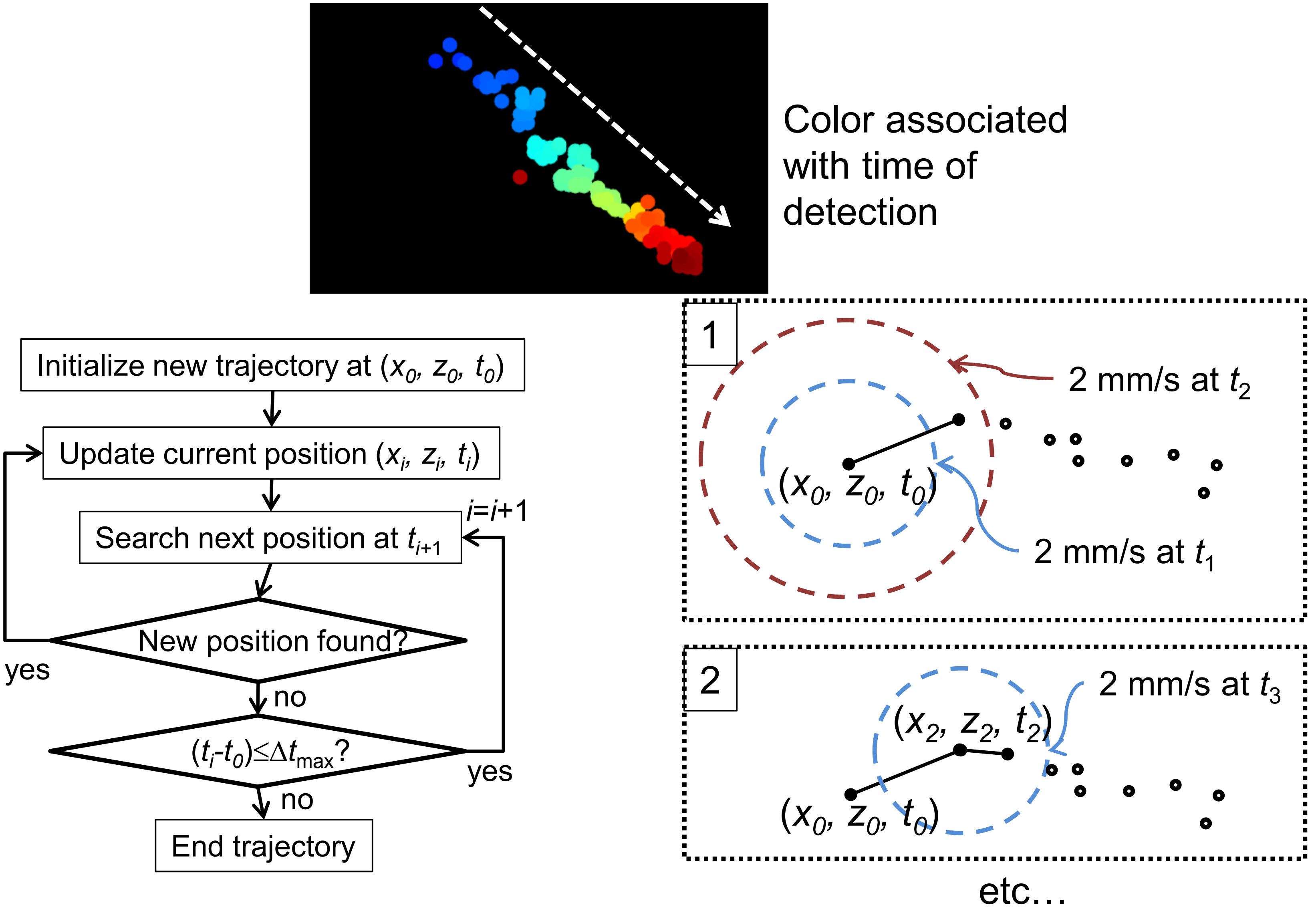

Supplementary Figure S10. Methods for tracking of individual MBs and estimation of the microvascular velocity. A set of positions was detected and the trajectory and velocity were estimated.

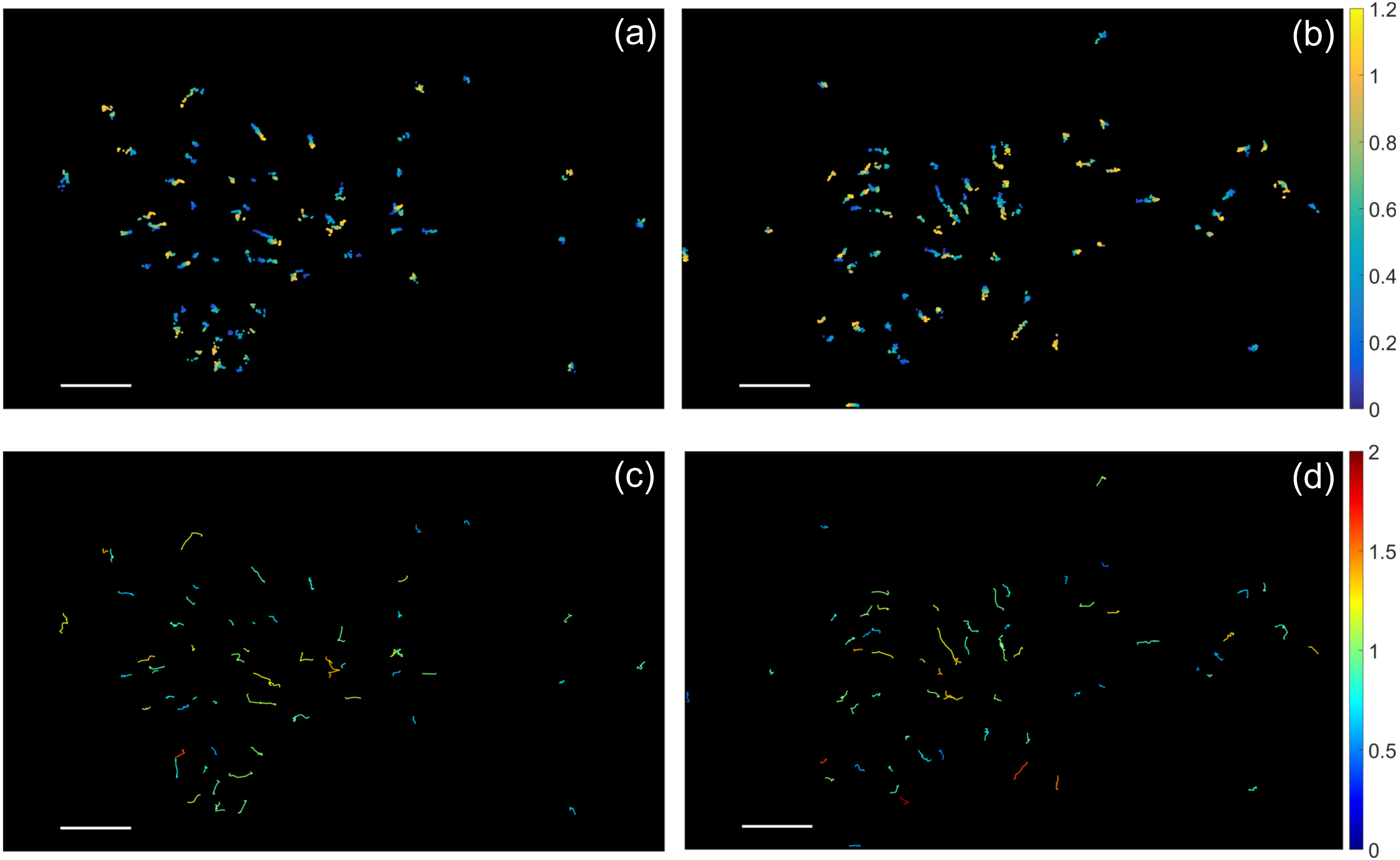

Supplementary Figure S11. Estimated trajectories from selected single cycles (between 2 successive respiration). (a) and (b) time of MB localization in cycle 21 (a) and 24 (b) (colorbar: time in seconds). (c) and (d) Estimated trajectories of isolated MBs with color coded velocity for cycle 21 (c) and 24 (d) (colorbar: velocity in mm/s). The scale bar represents 2mm.
